# Prevalence of oropharyngeal beta-lactamase-producing *Capnocytophaga *spp. in pediatric oncology patients over a ten-year period

**DOI:** 10.1186/1471-2334-5-32

**Published:** 2005-05-09

**Authors:** Anne Jolivet-Gougeon, Zohreh Tamanai-Shacoori, Laurent Desbordes, Virginie Gandemer, Jean-Louis Sixou, Nolwenn Morvan-Graveline, Michel Cormier, Martine Bonnaure-Mallet

**Affiliations:** 1 Equipe de Microbiologie, UPRES-EA 1254, Faculté des Sciences Pharmaceutiques et Biologiques, Université de Rennes 1, 2 avenue du Professeur Léon Bernard, 35043 Rennes, France; 2 Pediatric Oncology Department, CHU Hôpital Sud, 16 boulevard de Bulgarie, 35000 Rennes, France

## Abstract

**Background:**

The aim of this study was to evaluate the prevalence of beta-lactamase-producing *Capnocytophaga *isolates in young children hospitalized in the Pediatric Oncology Department of Hôpital Sud (Rennes, France) over a ten-year period (1993–2002).

**Methods:**

In neutropenic children, a periodic survey of the oral cavity allows a predictive evaluation of the risk of systemic infections by *Capnocytophaga *spp. In 449 children with cancer, 3,053 samples were collected by oral swabbing and plated on TBBP agar. The susceptibility of *Capnocytophaga *isolates to five beta-lactams was determined.

**Results:**

A total of 440 strains of *Capnocytophaga *spp. were isolated, 309 (70%) of which were beta-lactamase producers. The beta-lactamase-producing strains were all resistant to cefazolin, 86% to amoxicillin, and 63% to ceftazidime. The proportion of strains resistant to third-generation cephalosporins remained high throughout the ten-year study, while susceptibility to imipenem and amoxicillin combined with clavulanic acid was always conserved.

**Conclusion:**

These results highlight the risk of antibiotic failure in *Capnocytophaga *infections and the importance of monitoring immunosuppressed patients and testing for antibiotic susceptibility and beta-lactamase production.

## Background

*Capnocytophaga *spp. are capnophilic, gram-negative fusiform rods with gliding motility, common inhabitants of the oral cavity, but their role as an etiologic agent in juvenile periodontitis remains controversial [1]. In immunocompromised granulocytopenic patients, a number of complications, including septicemia, endocarditis, and peripheric lesions, have been reported [2-4]. Several authors have described cases of infections by *Capnocytophaga *strains resistant to antimicrobial agents [5-8]. An episode of bacteremia can be the consequence of bacterial translocation from oral flora. In the absence of bacterial strain isolation from blood cultures, empiric treatment might be adjusted according to the susceptibility of strains isolated during the survey. A sequential survey of the oral cavity during hospitalization was performed to evaluate the prevalence of beta-lactamase-producing *Capnocytophaga *isolates in children hospitalized in the Pediatric Oncology Department of Hôpital Sud (Rennes, France) over a ten-year period (1993–2002).

## Methods

Samples were collected by swabbing the bucco-pharyngeal area of children hospitalized in the Pediatric Oncology Department of Hôpital Sud (Rennes, France) [9]. Samples were taken periodically with a minimum of 15 days between each collection. All cancer patients from one to 17 years were included whatever the type of cancer, chemotherapy, and clinical situation were. The number of samples taken per child depended on the oncological disease (Acute Lymphoblastic Leukemia, Acute Myeloblastic Leukemia, others), the number of cures, and the number and length of hospitalizations or treatments. Each sample was inoculated on TBBP agar plates [4% trypticase soy agar supplemented with 5% sheep blood, 0.1% yeast extract (AES Laboratory, France), 100 μg/ml polymyxin and 50 μg/ml bacitracin (Sigma)] [10], which was then incubated for two to five days in a 10% CO_2 _atmosphere. Isolates were identified on the basis of colony morphology, Gram staining, negative catalase and oxidase reactions, API ZYM (BioMérieux, France) [11], and fatty acid profiles (gas chromatography, SHERLOCK Microbial Identification System™ MIDI Inc., Newark, DE, USA). Beta-lactamase production was tested using the qualitative chromogenic cephalosporin disk test (Cefinase^®^, BBL Microbiology Systems, Cockeysville, MD, USA). The results were read after 30 minutes.

Susceptibility testing was determined by standard methods and break points using the criteria of Bremmelgaard *et al*. [12] for screening determinations, and NCCLS [13] for intermediate/resistant strains. Minimal Inhibitory Concentrations (MICs) were confirmed by the E-test method (AES Laboratory, Combourg, France) using the same incubation conditions. Researchers tested the following antibiotics: amoxicillin, amoxicillin combined to clavulanic acid, cefazolin, ceftazidime, and imipenem.

## Results

Over the ten-year period of this study, researchers analyzed 3,053 samples from 449 hospitalized children (266 males and 183 females). *Capnocytophaga spp *(440 strains) were isolated in 232 children, on TBBP agar and identified with conventional methods. The annual percentage of children, who carried a *Capnocytophaga *strain at least once varied, with a minimum from 1995 to 1997 (17% and 22%, respectively), and maxima in 1993 (52%), 2001 (58%), and 2002 (61%). These results were also observed studying the number of *Capnocytophaga *isolates in the same periods: the number of *Capnocytophaga *isolates changed, with a minimum (11, 6%) in 1996 and a maximum (96, 22%) in 2001, without modification to the isolation and culture techniques (Table 1).

**Table 1:** Prevalence of *Capnocytophaga *spp. strains in periodic oral samples from children hospitalized in the Pediatric Oncology Department of Hôpital Sud (Rennes, France) from 1993 to 2002

**Year**	**Number of children included**	**Number of *Capno*^1 ^carriers^2 ^(%)**	**Number of samples collected**	**Number of *Capno *isolates (%)**
**1993**	60	31 (52)	305	56 (18)
**1994**	50	24 (48)	228	49 (21)
**1995**	45	10 (22)	195	14 (7)
**1996**	49	8 (16)	198	11 (6)
**1997**	58	9 (17)	251	13 (5)
**1998**	62	24 (39)	296	41 (14)
**1999**	68	24 (35)	267	38 (14)
**2000**	84	17 (20)	347	31 (9)
**2001**	85	49 (58)	429	96 (22)
**2002**	80	49 (61)	537	91 (17)
**Total**	**449**	**232 (52)**	**3,053**	**440 (14)**

^1^: *Capnocytophaga*

^2^: Number of children who were *Capnocytophaga *spp. carriers at least once during the studied period.

**Figure 1:**
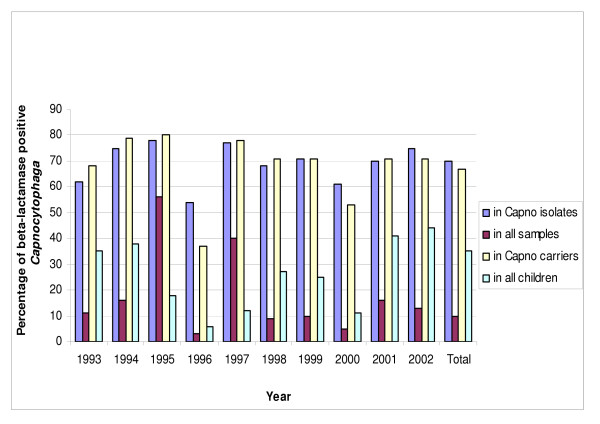
Prevalence of beta-lactamase producing Capnocytophaga spp. strains in periodic oral samples from children hospitalized in the Pediatric Oncology Department of Hôpital Sud (Rennes, France) from 1993 to 2002.

**Figure 2:**
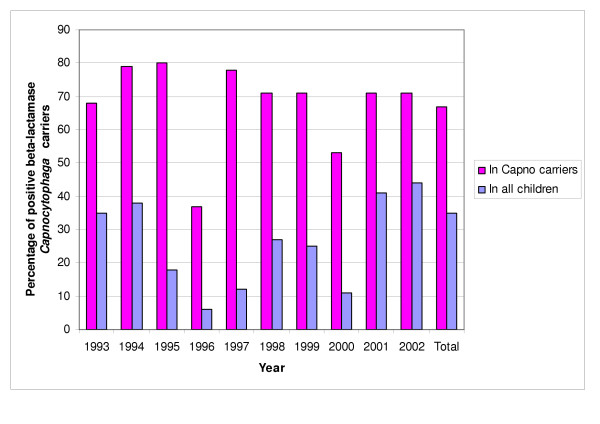
Percentage of positive beta-lactamase Capnocytophaga spp. carriers, from children hospitalized in the Pediatric Oncology Department of Hôpital Sud (Rennes, France) from 1993 to 2002.

Whatever the number of isolates, there was a high prevalence of beta-lactamase-producing strains (70%), with a varying incidence from 54% in 1996 (based on three out of 49 children) to 78% in 1995 (similarly, 11/14 isolates describes 8/45 children) (Figures 1 and 2). The susceptibility of *Capnocytophaga *strains was always conserved with imipenem (MIC < 4 μg/ml) and amoxicillin combined with clavulanic acid (MIC < 4/2 μg/ml). All beta-lactamase producing strains were uniformly resistant to cefazolin (MIC > 8 μg/ml), but the authors noted different levels of resistance to amoxicillin and ceftazidime (Table 2).

**Table 2:** Susceptibility of oral *Capnocytophaga *spp. isolated from 1993 to 2002 (Hôpital Sud, Rennes, France). Susceptibility testing was determined by standard methods and break points according to the criteria of NCCLS [13] and Bremmelgaard *et al*. [12]

**Year**	**Number of Resistant or Intermediate *Capnocytophaga *strains (%) (Total number of *Capno *strains = 440)**	
	
	Amoxicillin MIC^1^> 4 μg/ml	Ceftazidime MIC > 4 μg/ml
1993	31 (88)	28 (80)
1994	31 (84)	28 (75)
1995	11 (100)	11 (100)
1996	6 (100)	5 (83)
1997	10 (100)	9 (90)
1998	27 (96)	20 (71)
1999	23 (85)	10 (37)
2000	16 (84)	12 (63)
2001	54 (80)	38 (56)
2002	56 (81)	34 (49)
Total	265 (86)	195 (63)

Note: No strains were resistant to an imipenem and amoxicillin/clavulanic acid combination, and all beta-lactamase-producing strains were resistant to cefazolin.

^1^: Minimal Inhibitory Concentration (μg/ml).

## Discussion

In immunocompromised children, a periodic survey of the oral cavity during hospitalization allows a predictive evaluation of the risk of systemic infections by *Capnocytophaga *spp.

In this study, the authors correlated the number of *Capnocytophaga *isolates with the number of child carriers of *Capnocytophaga*, indicating that protocol was correctly conducted. A decrease in incidence was observed during the 1995–1997 period, which could be due to use, in first-line antibiotic protocols, of beta-lactamase inhibitor combinations. During the other time periods, extended-spectrum antibiotic were given. Aminoglycosides were always associated to beta-lactam antibiotics in all protocols from 1993 to 2002. Incidence rates of total extended-spectrum beta-lactamase producing bacteria (ESBL) in gram-negative rods responsible for infection in Hôpital Sud (Rennes, France), calculated for 1,000 days of hospitalization, varied in 2002 from 0.04 to 0.7 depending on the department (higher rate in reanimation units) . Interestingly, all *Capnocytophaga *spp. strains collected in this study appeared as colonizing strains, because neither bacteremia, nor other systemic infections, were observed during this study. Results of MIC determinations agree with previous works reporting that beta-lactamases confer a high degree of resistance to a wide range of beta-lactam antibiotics [14] while having no effect on imipenem and beta-lactamase-inhibitor combinations [15,16]. In a Canadian study, Roscoe *et al*. [17] reported that 36% of the strains collected mainly from clinical sources were beta-lactamase producers, while in a study in Taiwan, Lin *et al*. [3] reported that 18% of *Capnocytophaga *strains isolated from patients with bacteremia were beta-lactam resistant. In our study, the prevalence of beta-lactamase producers did not increase linearly during the study period.

A first hypothesis to explain these changes in incidence of beta-lactamase production could be the impact of current and previous therapy especially antimicrobial treatments or pathology, on the oral carriage of *Capnocytophaga *(study in progress). In a previous French study, Maury *et al*. [18] reported a high prevalence of beta-lactamase-producing *Capnocytophaga *species (75%), which they associated with previous beta-lactam treatments. Even if *Capnocytophaga *spp. belongs to the so-called late colonizers in the normal flora, meaning that it is more frequently found in children at 12 months and later, in a follow-up study of healthy infants from the age of 2 to 12 months, [19] Nyfors *et al*. reported a positive correlation between antimicrobial exposure and beta-lactamase production in oral anaerobic gram-negative species, while reporting only two beta-lactamase-producing *C. ochracea *isolates. In a recent contradictory study, [20]*Capnocytophaga spp*. has been detected in 1.9% of all episodes of fever and neutropenia before antibiotic therapy, *versus *0.3% during antimicrobial treatment.

A second hypothesis to explain these changes in incidence of beta-lactamase production could be linked to the immediate environment and close contact. These conditions have a great influence on the composition of the flora, and coming and going between home and hospital could modify the children's oral ecosystem. A turnover of the bacterial population from beta-lactamase-positive to beta-lactamase-negative strains may occur in young children with a developing oral ecosystem.

Another explanation of this high prevalence of beta-lactamase production could be conferred on transfer of encoding-resistance genes. Anaerobic bacteria are known to be able to exchange genetic material with aerobic bacteria, even though antibiotic-resistance genes are expressed differently in aerobic and anaerobic bacteria [21]. The spread of antibiotic resistance can also play a great part in nosocomial infections in neutropenic patients [22]. The high rate of resistance to third-generation cephalosporins observed in this study could be due to the dissemination of an epidemic clone. Some ceftazidime-resistant strains have the same susceptibility and plasmid profiles (data not shown). Other authors have described resistant clinical isolates [5,6,14,16], but Rosenau *et al*. [8] were the first to characterize a plasmid-encoded TEM extended-spectrum beta-lactamase in *Capnocytophaga spp*. Clavulanate-sensitive cephalosporinases belonging to class A group 2e from the classification of Bush *et al*. [23] have recently been described [7,24]. They differ by only one or two substitutions in their sequences (*cfx*A, Genbank accession No. U75371; *cfx*A2, Genbank accession No. AF118110; *cfx*A3, Genbank accession No. AF472622). Large transposons encode all the functions needed for their own conjugation and for resistance to antimicrobial agents, including third-generation cephalosporins.

## Conclusion

These results show the importance of testing for the antibiotic susceptibility of and beta-lactamase production by clinical *Capnocytophaga *strains, in which beta-lactamase production has become very common. In neutropenic patients, resistance to third-generation cephalosporins should be taken into consideration upon hospitalization to adapt the empiric antimicrobial treatment previously dispensed.

## Competing interests

The author(s) declare that they have no competing interests.

## Authors' contributions

AJG carried out the microbiological studies and wrote the manuscript. ZTS, LD and NMG participated in the microbiological studies. VG was in charge of the clinical studies. Provision of advice was given by JLS, MC and MBM. All authors read and approved the final manuscript.

## Pre-publication history

The pre-publication history for this paper can be accessed here:


